# Comparison of 1-Site and 2-Site Phacotrabeculectomy in the Small Adult Eyes With Concomitant Cataract and Glaucoma

**DOI:** 10.1097/MD.0000000000002405

**Published:** 2016-02-08

**Authors:** Xiaobo Xia, Ying Tian, Zhenkai Wu, Dan Wen, Weitao Song

**Affiliations:** From the Department of Ophthalmology, Xiangya Hospital, Central South University, Changsha, China.

## Abstract

The aim of this study was to compare the outcomes after phacotrabeculectomy at 1 or 2 sites in the small adult eyes with concomitant cataract and glaucoma.

Patients who had 1-site (n = 26) or 2-site (n = 14) phacotrabeculectomy over a 4-year period at an eye surgery center were included. Eighteen eyes of 18 patients with glaucoma using any 1 prostaglandin analogue (latanoprost, travoprost, or bimatoprost) were compared with 8 normal control patients. The records of patients were reviewed, and intraocular pressure, best-corrected visual acuity, axial length, anterior chamber depth, corneal endothelial cell (CEC) density, Diopter were measured. The outcome was compared with postoperative and preoperative measurements for 3-month follow-ups.

The follow-up time was 3 months. There was no difference between the operations in improving best-corrected visual acuity, lowering intraocular pressure, shortening axial length, and deepening anterior chamber depth. However, 2-site surgery was associated with significantly more CEC loss and refractive error. Postoperative complications were not different between the 2 groups.

The CEC loss and the refractive error in 2-site group were higher than that of 1-site group. One-site surgery seems to cause less CEC damage and refractive error than the 2-site operation during the follow-up time of 3 months.

## INTRODUCTION

The small adult eye phenotypes include anophthalmos, nanophthalmos, and microphthalmos, and the latter 2 are synonymous and could be divided into simplex and complex microphthalmos according to ocular or systemic abnormalities.^1–4^ Currently, few data are available on the prevalence of small adult eye phenotypes. The estimated prevalence for microphthalmos is 0.002% to 0.017% worldwide and 0.009% in China.^5^ It was suggested that patients with simple microphthalmos consist of 0.05% to 0.11% of ophthalmic patients.^6^ Up to now, there is still no consensus on the definition of nanophthalmos and microphthalmos, and axial length (AL) <21 mm is generally accepted as the definition of nanophthalmos/microphthalmos. Diagnostic features include a small eye, short AL, hypermetropic, microcornea, shallow anterior chamber, narrow angle, large crystalline lenses, high incidence of angle closure glaucoma, thickened sclera, choroidal congestion, uveal effusion, and serous retinal detachment.^7–10^

Angle closure glaucoma and cataract are both common comorbidities among the small adult eye. Nanophthalmos/microphthalmos is associated with significant visual morbidity owing to angle closure glaucoma and lens opacity, and needs treatment by phacotrabeculectomy.^11–13^ Nevertheless, phacotrabeculectomy in nanophthalmic/microphthalmos eyes has high incidences of intraoperative and postoperative complications. Surgical manipulation in a narrow and crowded anterior chamber with increased vitreous pressure is challenging, which always tends to cause pupillary blockage. During surgery, a sudden decrease in intraocular pressure (IOP) may result in uveal effusions, secondary retinal detachment, vitreous hemorrhage or malignant glaucoma, and even loss of the affected eye. Despite several potential complications, surgical outcomes for small eyes have been encouraging with the advances in phacotrabeculectomy and intraocular lens.^14–18^

In this retrospective, consecutive, case–control series study, IOP, keratometry, corneal endothelial cells (CECs), best-corrected visual acuity (BCVA), and intraoperative and postoperative complications were reviewed in patients with small adult eye and concomitant cataract and glaucoma who underwent 1-site and 2-site phacotrabeculectomy. We aimed to evaluate 2 surgery techniques in patients who have had cataract extraction with foldable intraocular lens implant (IOL) and trabeculectomy to better aid in the process of selecting appropriate preoperative therapy approaches.

## METHODS

### Participants

Forty small adult eyes of 24 patients that met the eligibility criteria were enrolled from patients at our hospital between March 1, 2010, and March 31, 2014. Inclusion criteria were based on clinical diagnosis of nanophthalmos/microphthalmos and ocular surgery for cataract and angle closure glaucoma. The inclusion criteria were defined as corneal diameter (CD) <11 mm, anterior chamber depth (ACD) <2.2 mm, and AL <21 mm.^7,8^ Exclusion criteria included corneal opacities, corneal endothelial abnormalities, posterior segment disorders, cataracts not suitable for phacoemulsification, and increased risk of endophthalmitis such as active adnexal and ocular surface infection. All enrolled patients received simultaneous phacoemulsification and trabeculectomies at either 1-site (n = 26, 15 patients) or 2-site (n = 14, 9 patients), and pre- and postoperation assessments of the eyes were performed as described previously.^19^

### Surgery Procedure

Phacotrabeculectomy and posterior chamber foldable IOL implantation were performed by the same experienced surgeon. Before the operation, the pupil was dilated using 1.0% tropicamide and 2.5% phenylephrine (Sante, Osaka, Japan), and the eyes received topical anesthesia by using 0.5% proparacaine hydrochloride and 4% lidocaine. One-site group had a trabeculectomy in a superior scleral flap (size: 3 mm × 4 mm, thickness: 1/2 sclera) combined with phacoemulsification through the same incision; 2-site group had a separate, superior scleral flap (size: 3 mm × 4 mm, thickness: 1/2 sclera) for trabeculectomy and clear corneal incision for phacoemulsification. The incision for phacoemulsification was made by stab knife (Sharper, Middleboro, MA, USA), and 1% sodium hyaluronate (Healon, Kalamazoo, MI, USA) was injected into the anterior chamber. Next, phacoemulsification was performed and the residual cortex was cleared by using an irrigation/aspiration system. The lens capsule was inflated with Healon, and the foldable IOLs (Reyner, East Sussex, UK) were implanted in the capsular bag. The scleral flap was sutured 2-pin with 10-0 Nylons (Alcon, Fort Worth, TX, USA) and conjunctiva wound was sutured with 8-0 absorbable catgut (Alcon, Fort Worth, TX, USA).

### Statistical Analysis

Statistical analyses were performed using SPSS 17.0 software (SPSS, Chicago, IL). Differences among 1-site and 2-site phacotrabeculectomy were assessed by Kruskal–Wallis test for continuous parameters and Fisher exact test for numeric parameters. *P* values of less than 0.05 indicated significant differences.

## RESULTS

Forty small adult eyes of 24 patients (21 women, 3 men) who were enrolled into this study between March 1, 2010, and March 31, 2014, met the inclusion criteria for the definition of nanophthalmos/microphthalmos and diagnostic criteria of angle closure glaucoma and cataract. Thirty-two eyes of 16 patients were diagnosed as bilateral angle closure glaucoma accompanied by cataracts. Preoperatively, 8 eyes of 8 patients were unilateral angle closure glaucoma and cataract. IOP of all nanophthalmic/microphthalmos eyes were controlled below 25 mm Hg with medicines preoperatively. Ten patients have undergone bilateral 1-site phacotrabeculectomy, and bilateral 2-site phacotrabeculectomy was performed in 6 patients. Phacotrabeculectomy and bag Posterior chamber intraocular lens (PCIOL) implantation were performed using the above-mentioned technique.

The demographics of the eyes with 1-site group and 2-site group are summarized in Table 1. The mean age of the enrolled patients in 1-site group was 56.68 years (range 55–78 years), while that of 2-site group was 58.46 years (range 53–76 years). The mean age of 1-site group was younger than the patients of 2-site group. However, a significant difference in mean age between the 2 groups was not detected. The proportion of female subjects was 78.3% in 1-site group and 76.9% in 2-site group (*P* = 0.02). These results showed that the prevalence of female was higher than that of male.

**TABLE 1:**
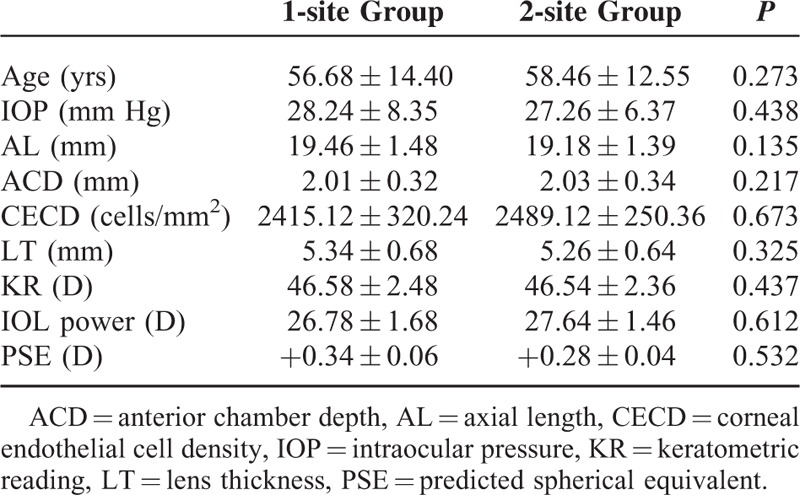
Preoperative Data

All of the eyes in 2 groups underwent antiglaucomatic medical treatment. Preoperatively, the mean IOP was 28.24 mm Hg (range 23–38 mm Hg) in 1-site group and 27.26 mm Hg (range 22–41 mm Hg) in 2-site group. The mean AL and mean ACD was 19.46 mm (range 16.22–19.90 mm) and 2.01 mm (range 1.76–2.24 mm), respectively, in 1-site group, and was 19.18 mm (range 16.46–19.68 mm) and 2.03 mm (range 1.64–2.12 mm), respectively, in 2-site group. Although the cornea diameter of the nanophthalmos/microphthalmos was small, CEC density was not different form the normal eyes (1-site group: 2415.12/mm^2^; 2-site group: 2389.12/mm^2^). The mean lens thickness was 5.34 mm (range 5.16–5.58 mm) in 1-site group and 5.26 mm (range 5.12–5.74 mm) in 2-site group, compared with less than 5 mm in normal eyes. The CD of the nanophthalmos/microphthalmos was usually less than 10 mm. The minimum and maximum CD in 2 groups was 8.00 and 9.80 mm, respectively, and the average values were 8.90 mm. According to the keratometric reading (KR) and AL in 2 groups (mean KR: 46.58 D, range 43.14–49.48 D, 1-site group vs mean KR: 46.5 D, range 43.35–49.52 D, 2-site group), the mean PCIOL power was 27.78 D (range 24.50–29.50 D) versus 27.64 D (range 25.50–29.00 D) and mean predicted spherical equivalent was +0.34 D (range +0.012 to +0.48 D) versus +0.28 D (range +0.016 to +0.35 D) in 1-site group and 2-site group, respectively. There were no significant differences in these data between 1-site group and 2-site group.

Intraoperatively, 1 of 26 eyes (3.85%) in 1-site group was switched to extracapsular cataract extraction because of crowded and shallow anterior chamber. Clear corneal incision was sutured because of the leakage in 3 eyes of 2-site group (21.43%) and corneal stroma edema occurred at incision in 2-site group (5 of 14 eyes, 35.71%). There were no other complications in 2 groups.

All patients received examination with slit lamp, IOP, and VA on the day after surgery. Five eyes of 1-site group (19.23%) and 2 eyes of 2-site group (14.29%) had mild corneal edema and the remaining were completely transparent. In 2 groups, exudation and Tyndall phenomenon was negative or weakly positive in anterior chamber, and each IOL was properly located in the capsular bag of posterior chamber. Twenty-four eyes (92.31%) had increased uncorrected visual acuity from 2 to 8 lines on the Snellen chart in 1-site group, and the remaining 2 eyes (7.69%) in 1-site group had no change with uncorrected visual acuity. In 2-site group, 9 of 14 eyes (64.29%) had increased uncorrected visual acuity from 3 to 6 lines on the Snellen chart; 2 of 14 eyes (14.29%) had decreased uncorrected visual acuity from 1 to 2 lines, and the remaining 3 eyes (21.43%) had no change. IOP was detected with Goldmann tonometer from 6 to 16 mm Hg in 2 groups.

Table 2 summarizes preoperative and postoperative BCVA in 2 groups about 3 months after surgery. BCVA was improved postoperatively in 1-site group and 2-site group, with significant differences (0.23 ± 0.20 vs 0.45 ± 0.12; 0.20 ± 0.08 vs 0.52 ± 0.14; *P* = 0.005 and 0.01; respectively). These results showed that BCVA was improved to the same extent by 2 different surgical methods.

**TABLE 2:**
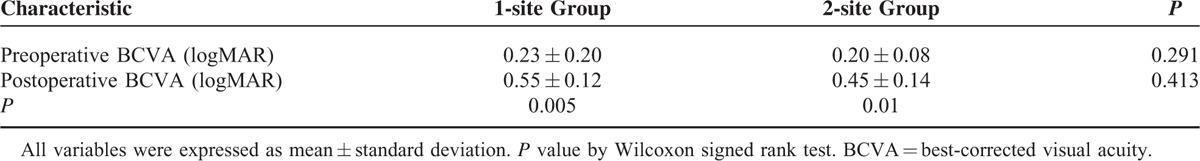
Preoperative and Postoperative BCVA

As summarized in Table 3, 3 months after surgery, mean IOP was 14.65 ± 3.67 mm Hg (range 9–17 mm Hg) in 1-site group and 15.16 ± 3.35 mm Hg (range 10–19 mm Hg) in 2-site group, with no significant difference between the 2 groups. However, preoperative IOP and postoperative IOP showed significant difference (*P* = 0.001). Postoperatively, mean AL was shortened 0.54 mm (1-site group) and 0.93 mm (2-site group), and significant difference between 1-site group and 2-site group was detected. AL was shortened much more in 2-site incision than 1-site incision. Mean ACD was deepened 1.99 mm in 1-site group and 1.92 mm in 2-site group, with no significant differences between 1-site group and 2-site group. The mean CEC density was decreased from 2415.12 to 2387.34/mm^2^ in 1-site group and from 2389.12 to 1867.13/mm^2^ in 2-site group. The results showed that phacotrabeculectomy using 2-site technique led to more CEC loss than 1-site technique (*P* = 0.001). In addition, there was a significant difference in refractive error between 1-site group and 2-site group postoperatively, and 2-site phacotrabeculectomy led to more refractive errors and astigmatism than 1-site technique.

**TABLE 3:**
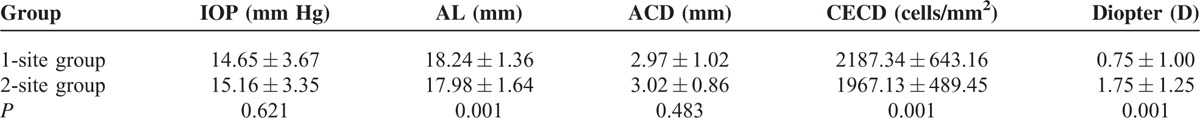
Postoperative Data

## DISCUSSION

Currently, birth prevalence of microphthalmos is 0.002% to 0.017%, and the prevalence of simple microphthalmos in ophthalmic patients is 0.05% to 0.11%.^4,16,18^ Nanophthalmos is known to be related to a high risk of angle closure glaucoma, and nanophthalmos and angle closure glaucoma exhibit similarity such as short AL, small radius of corneal curvature, shallow anterior chamber, and thick crystalline lens.^5,6^ Cataract of small eyes is formed gradually with the increase of age, and angle closure glaucoma could increase the incidence of cataract. Therefore, phacotrabeculectomy has to be performed.

Phacotrabeculectomy can be performed via 2 approaches: using 1-site for trabeculectomy and phacoemulsification; or using 2 sites with a prelimbal filtering incision for trabeculectomy and a clear cornea incision for phacoemulsification, respectively.^19^ Nevertheless, phacotrabeculectomy in small eyes could cause high incidence of complications such as uveal effusions, secondary retinal detachment, vitreous hemorrhage, or malignant glaucoma, perhaps due to sudden decrease of IOP.^8,9^ Furthermore, trabeculectomy and cataract incision could impact keratometry readings.^20^^.^^21^

In this retrospective study, we found that both 1-site and 2-site techniques led to significantly shortened AL, consistent with previous reports that AL was significantly decreased following trabeculectomy.^22–24^ In addition, we compared the effects of 1-site versus 2-site phacotrabeculectomy on CEC density, and found that 1-site procedure led to less central CEC loss and less central CEC area expansion, perhaps due to a more posterior approach employed in 1-site phacotrabeculectomy. A previous study compared phacoemulsification through a scleral tunnel and phacoemulsification through a clear corneal incision and showed that the former led to less postoperative endothelial damage.^25^

The overall mean CEC loss was 15.7%, which may result in a CEC density of less than 500 cells/mm^2^, which has been reported as the threshold for endothelial decompensation.^26^ It was suggested that CEC density would be stabilized 3 months after cataract extraction.^27^ In current study with 3 months follow-up, the mean CEC loss rate was 9.44% in 1-site group and 20.97% in 2-site group, indicating that 1-site surgery seems to cause less CEC damage than 2-site surgery. One-site surgery may be a better option for managing coexisting visually significant cataract and uncontrolled glaucoma in patients with small eyes and higher risk of endothelial damage. Our data are consistent with a previous study showing the correlation between shorter AL and higher risk of CEC loss.^28^

In summary, when small adult eyes with concomitant cataract and glaucoma underwent phacotrabeculectomy, CEC loss and the refractive error in 2-site group were higher than in 1-site group. Therefore, nanophthalmos/microphthalmos patients who have had cataract extraction with foldable IOL and trabeculectomy should be performed with 1-site surgery approach preoperatively.
